# Study on genes of the serotonergic system and suicidal behavior: protocol for a case–control study in Mexican population

**DOI:** 10.1186/1471-244X-14-29

**Published:** 2014-02-05

**Authors:** Carlos Alfonso Tovilla-Zárate, Thelma Beatriz González-Castro, Isela Juárez-Rojop, Sherezada Pool García, Martha Patricia Velázquez-Sánchez, Mario Villar-Soto, Alma Genis, Humberto Nicolini, María Lilia López-Narváez, María Antonia Jiménez-Santos

**Affiliations:** 1 División Académica Multidisciplinaria de Comalcalco, Ranchería Sur, Cuarta Sección, CP 86650 Comalcalco, Tabasco, México; 2 Universidad Juárez Autónoma de Tabasco, División Académica de Ciencias de la Salud, Villahermosa, Tabasco, México; 3 Hospital General de Comalcalco, Tabasco, Secretaría de Salud, Comalcalco, Tabasco, México; 4 Hospital de Alta Especialidad “Gustavo A. Rovirosa P”, Villahermosa, Tabasco, México; 5 Instituto Nacional de Medicina Genómica (INMEGEN), Servicios de Atención Psiquiátrica (SAP), Secretaría de Salud, México, DF, México; 6 CIGEN, Centro de Investigación Genómica, Comalcalco, Tabasco, México; 7 Hospital General de Yajalón, Yajalón, Chiapas, México

**Keywords:** Suicide, Serotonin receptor, Association, Mexican population

## Abstract

**Background:**

Suicidal behavior is a leading cause of injury and death worldwide. Several studies have provided a possible relationship between genetic factors and suicidal behavior. Also, these studies have shown evidence for altered serotonergic neural transmission in the pathogenesis of suicidal behavior. In addition, genes pertaining to the serotonergic system have been proposed as candidates to establish biological correlates between suicidal behavior and the serotonergic system. The most studied genes are SCL6A4, HTR2A, HTR2C, HTR1A, HTR1B, TPH-1, and TPH-2. To get a comprehensive understanding of the association with suicidal behavior we will conduct genotype assays studies in a Mexican population.

**Methods/Design:**

We will conduct a case–control study. The population sample will comprise adolescent and adult patients admitted for attempted of suicide and diagnosed by a psychiatrist. A peripheral blood sample will be taken from all the subjects (cases and controls). Genomic DNA from the leukocytes blood sample will be extracted. The genotypes of interest are distributed in the following genes: SCL6A4, HTR2A, HTR1A, HTR1B, HTR2C, TPH-2 and TPH-1. All the samples will be analyzed using a polymerase chain reaction (PCR) end-point method. We will evaluate the Hardy-Weinberg Equilibrium. The chi-squared test or Fisher’s exact test will be used to compare genotype and allele frequencies between control and case groups. The Quanto 1.2 software will measure the sample size of the association. For all the association analyses the level of significance will be set at *p* = 0.05 and the confidence interval at 95%.

**Discussion:**

Suicidal behavior has been increase in Mexico, principally in young population. Our study will demonstrate the association between serotoninergic genes and suicide behavior in Mexican population.

## Background

Suicide is defined as the act of killing oneself in a voluntary way. The World Health Organization (WHO) classifies this action as a deliberate self-harm against oneself [1]. Suicidal behavior (SB) includes from just the thought of planning the suicide, attempt to kill oneself, to the consummation of the act [2,3]. WHO estimates that suicide represents at least 2% of the deaths in the world [1,4]. According to some statistics people aged 75 or older resort to suicide three times more often than youngsters. However, suicide among young people between 12 and 25 years old has been on the rise around the world [5]. Compared to other countries, Mexico in 1980–1999 exhibited an increase in suicide of 90.3% in men and 25.5% in women [4,6-8]. Moreover, in the period 1990 to 2000 the increase was 150% in the young population [6,9], and according to a recent statistic in 47% of the individuals between 14 and 19 years old suicide is the second cause of death among the young Mexican population. In particular the state of Tabasco occupies the third place in these figures and the first in the southeastern region of the country of patients who presented suicidal behavior [5,8].

Suicide is considered a multifactorial event that involves biological, social, individual and environmental factors [10]. Suicidal behavior has been associated among many risk factors with psychiatric disease, such as anxiety disorder, major depression, substance abuse (alcohol and drugs), personality disorder and schizophrenia [11]. Other risk factors for suicidal behavior are marital status, unemployment, adverse events, child abuse and neurological disease [12]. However, sociocultural events do not provide a complete explanation for suicidal behavior. As a result it has been accepted that suicide will be fully understood when the interaction between biological and environmental factors are taken into consideration [13]. One factor that has been involved in suicidal behavioral is genetic risk. This evidence is based on genetic epidemiology studies that include twin [14], family [10,15] and adoption studies [16].

The genes highlighted in the association with suicidal behavior are those that encode proteins of the serotonergic system. Moreover, low 5-hydroxyindoleacetic acid concentration (5-HIIA) in cerebrospinal fluid of depressed suicide attempters and in brain stems of completed suicides has been reported [17-19].

The main genes implicated in suicidal behavior are associated with serotonin production and its transporter. The serotonin transporter (SLC6A4) has been the most studied in relation to SB. This gene is located in region 17q11.1-17q12 [20]. One of the polymorphisms of SLC6A4 is 5-HTTLPR, situated in the promoter region and characterized by the 44 bp insert (alleles I)/ deletion (allele s) [21,22]. The expression of this gene is regulated by elements in the promoter region. The literature describes that the homozygous genotype to allele I shows more transcriptional activity than genotypes with one or two ss alleles [22-25]. This finding led to an association between the s allele polymorphism and anxiety disorder, depression and suicidal behavior, among other diseases [26-28]. Recently, a meta-analysis of 44 studies reported that carriers of the short allele exhibited increased risk for suicidal behavior [29].

Other genes studied are serotonin receptors, mainly HTR2A, HTR1B and HTR2C. Several reports have analyzed the possible association of C102T HTR2A polymorphism with SB and have observed an excess of the C/C genotype in patients with suicide attempts [29-31]. Similarly, the decreased number of 5-HT1B receptors in the prefrontal cortex in patients with SB locates this receptor as a candidate gene study [32]. The receptor 5-HT2C has been studied in several Caucasian and Asian populations with significant association [33,34].

Other genes that may play a role in SB pathogenesis are tryptophan hydroxylase (TPH) and monoamine oxidase A (MAO-A) [33]. The TPH gene has two known isoforms: TPH2, which is expressed in brain and TPH1, expressed mainly in the periphery. To date there are more than 30 reports of these two isoforms in association with SB. With regard to MAO-A, most studies have identified a VNTR in the promoter region, which is correlated with suicide victims [35]. On the other hand, the literature also suggests that epigenetic alterations in specific genes are associated with SB, as has been observed in other psychiatric diseases. In addition, DNA methylation can influence gene expression in neurons and glial cells. As a result, alterations in personality traits have been described [20,36,37].

### Aims and objectives

This article focuses on the analysis of SB in the Mexican population, particularly in the Tabasco state. The aim of this study is to determine the association of several serotonergic genes (SCL6A4, HTR2A, HTR2C, HTR1A, HTR1B, TPH-1, and TPH-2) with suicidal behavior in Mexicans.

## Methods/Design

### Study patients

The population sample chosen for the study will comprise adolescent and adult patients (14–60 years old) admitted to the Regional Hospital of Comalcalco, Tabasco and Hospital “Gustavo A. Rovirosa P” and diagnosed with SB by a psychiatrist. We expect to recruit 500 cases. The inclusion/exclusion criteria will be the following: participants must be Mexican subjects descending from Mexican parents and grandparents, current substance abuse, history of substance dependence, history of bipolar disorder, concomitant medical or neurological illness, and intellectual disability. Also, the authorized control group will consist of physically healthy subjects on medical evaluation, with no manifested psychiatric problems, as assessed in brief interviews by psychiatrists; subjects will be recruited from the blood donor center of the same hospital. We expect to recruit 500 controls. SB diagnosis will be based on the DSM-IV instrument; disorders pertaining to Axis I, II and III will be considered. For the control group, a systematic interview will serve to obtain a detailed medical and psychiatric history. The time frame the recruitment of cases-controls will be November 2012 to November 2015.

### Ethics statement

Written informed consent will be obtained from all subjects after they are given a detailed and extensive description of the study; they will not receive any economical remuneration. The study was approved by two Ethics Committee (Hospital General de Comalcalco and Hospital Dr. Gustavo A. Rovirosa Perez) and Research Committee of the university the Tabasco, México (DAMC-UJAT). Also, the study will be carried out in accordance with the ethical standards convened in the 1964 Declaration of Helsinki.

### Genotype assays

A peripheral blood sample will be taken from all the subjects (cases and controls). Genomic DNA from the leukocytes blood sample will be extracted using a modified version of the protocol by Lahiri [38-40]. The genotypes of interest are distributed in the following genes: SCL6A4 (HTTLPR, rs25531, rs6355), HTR2A (rs6313, rs6311, rs6314), HTR1A (rs6295, rs1423691, rs878567), HTR1B (rs6296), HTR2C (rs547536, rs2192372, rs4272555, rs6318, rs2428707), TPH-2 (rs7305115) and TPH-1 (rs179913, rs1800532). All the samples will be analyzed using a polymerase chain reaction (PCR) end-point method. The final volume of the PCR reaction for HTR2A, HTR1A, HTR2C, TPH-2 and TPH-1 will be 5 μL and will consist of 20 ng genomic DNA, 2.5 Fluorescence Labeling (FL) TaqMan Master Mix, and 2.5 FL 20x Assay. The amplification will be performed in 96-well plates using the TaqMan Universal Thermal Cycling Protocol. After the PCR endpoint procedure, fluorescence intensity will be measured with the 7500 Real-Time PCR system using SDS v2.1 software (Applied Biosystems). All genotyping will be carried out blind to patient outcome. As a quality control in our genotyping analyses we will make random blind duplicates. In the case of the SCL6A4 gene, we selected the 5HTTLPR polymorphism. The triallelic system will be analyzed in two steps. First, to genotype s and l alleles, a region encompassing 5HTTLPR will be amplified, using the primers by Hilss et al. [41]. In the second step, fragments of La and Lg alleles will be identified using the 5′exonuclease assay (TaqMan SNP genotyping assay-by design) [42].

### Statistical analysis

Demographic factors will be evaluated with the statistics program SPSS v.16. In addition, we will evaluate the Hardy-Weinberg Equilibrium (HWE) using Pearson’s goodness-of-fit chi-squared test. The p value will be corrected according to the number of comparisons made in each locus. Also, the chi-squared test or Fisher’s exact test will be used to compare genotype and allele frequencies between control and case groups. The Quanto 1.2 software will measure the sample size of the association. For all the association analyses the level of significance will be set at *p* = 0.05 and the confidence interval at 95%.

## Discussion

Suicide is a major public problem around the world. Reports from the World Health Organization (WHO) indicate that suicide accounts for the largest share of self-harm in developed countries. Suicide is foreseen as an even greater contributor to the global burden of disease over the coming decades [5-7]. In Mexico, it is one of the five leading causes of death in 34 years old and the third in individuals between 15 and 24 years of age. Previous studies have reported that between 2.5% and 4.3% of the Mexican population have presented a suicide attempt at some time in their lives. In 2008, in Mexico, 6 601 210 inhabitants had experienced suicidal ideation [4,8,43,44]. Because the presence of psychiatric illnesses, such as anxiety disorders, depression, substance abuse (alcohol and drug dependence), personality disorders, schizophrenia and panic disorder do not fully explain suicidal behavior, a role for a genetic basis has been suggested in the pathology of SB [45,46]. In association studies, the most studied genes are those encoding proteins involved in the metabolism of serotonin such as tryptophan hydroxylase (TPH 1 and TPH 2), serotonin transporter (5-HTT), 5 mono-amine-oxidase (MAO-A), as well as the serotonin receptors, especially HTR1A, HTR1A, HTR2A and HTR2C. Although several genetic association studies have been undertaken in populations worldwide, progress has been slow since the predisposition to commit suicide does not follow a common Mendelian inheritance pattern.

Suicidal behavior has increased substantially in Mexico, and this rise can not be explained solely by environmental factors. Although there are several reports on this issue in Mexico, to date there are no studies evaluating the role for a serotonergic pathway in association with suicidal behavior. Genetic research conducted in Mexico will try to establish the regulation of genes in the pathology of SB.

## Abbreviations

WHO: World Health Organization; SB: Suicidal behavior; 5-HIIA: 5-hydroxyindoleacetic acid concentration; HTR2A: Serotonin 2A receptor; HTR2C: Serotonin 2C receptor; HTR1A: serotonin 1A receptor; HTR1B: Serotonin 1B receptor; TPH-1: Tryptophan hydroxylase 1; TPH-2: Tryptophan hydroxylase 2; HWE: Hardy-Weinberg Equilibrium.

## Competing interests

The authors declare that they have no competing interests.

## Authors’ contributions

TZC and JRI conceived the study, participated in the design and helped to draft the manuscript. PGS, VSMP and VSM helped to select the clinical criteria for patients. GA, NH, GCTB and MLLN helped to select the genes and polymorphisms in the study. TZCA, GCTB and JSMA wrote the draft of the manuscript. All authors read, critically revised, and approved the final version of the manuscript.

## Pre-publication history

The pre-publication history for this paper can be accessed here:

http://www.biomedcentral.com/1471-244X/14/29/prepub
